# Environmental factors associated with invasive mold infections at a tertiary-care hospital

**DOI:** 10.1017/ash.2023.349

**Published:** 2023-09-29

**Authors:** Lindsey Tully, Schuyler L. Gaillard, Lucy Zheng, Tara Millson, Princy Kumar, Joseph Timpone

## Abstract

**Background:** Invasive mold infections (IMIs) in hospitalized patients can result in significant morbidity and mortality. Environmental factors, such as hospital construction and negative air-pressure rooms (NAPRs), have been associated with hospital-acquired IMI. Increased utilization of NAPRs during the COVID-19 pandemic created a unique opportunity to examine the impact of NAPRs on IMI risk. **Methods:** From 2018 to present, a new pavilion was being constructed adjacent to our hospital. The Theradoc platform was used to identify positive mold cultures among adult patients hospitalized at our institution between March 1, 2017, and October 15, 2022. We performed a retrospective chart review of 262 mold isolates to determine patient demographics, timing of IMI, and their relationship to hospital construction and exposure to NAPR. IMI incidence was compared across 3 observation periods: (A) before hospital construction; (B) during hospital construction alone; and (C) during hospital construction and NAPR enhancement during the COVID-19 surge. Hospital-acquired IMI was defined as an infection that occurred >72 hours after admission. A REDCap database was created for data storage and R software was used for data analysis. **Results:** Of the 262 mold isolates identified, 61 cases were classified as IMI, of which 29 were hospital-acquired IMI. The mean age of IMI patients was 51.8 years, and 55.2% were male. Among them, 20.7% were exposed to NAPR during admission; 65.5.% were immunocompromised; and 2 patients were diagnosed with COVID-19. The all-cause mortality rate among hospital-acquired IMI cases was 79.3% (23 of 29). Also, 82.8% of hospital-acquired IMI cases were respiratory in nature, with 83.3% of these cases due to *Aspergillus* spp. Yearly rates of hospital-acquired IMI were 3.0 before construction versus 5.6 during construction (periods B and C). Yearly rates of hospital-acquired IMI, respiratory IMI, and invasive pulmonary aspergillosis by period were as follows: Period A had 3 hospital-acquired IMI cases per year, 2 hospital-acquired respiratory IMI cases per year, and 3 hospital-acquired invasive pulmonary aspergillosis cases per year. Period B had 4.5 hospital-acquired IMI cases per year, 3.5 hospital-acquired respiratory IMI cases per year, and 3.0 hospital-acquired invasive pulmonary aspergillosis cases per year. Period C had 6.5 hospital-acquired IMI cases per year, 5.4 hospital-acquired respiratory IMI cases per year, and 5.0 hospital-acquired invasive pulmonary aspergillosis cases per year. **Conclusions:** Hospital-acquired IMI was associated with a high mortality. Our data demonstrate a >2-fold increase in yearly incidence of hospital-acquired IMI before construction compared with during construction in association with increased implementation of NAPR. We have now reversed the trend in NAPR at our hospital’s designated COVID-19 units.

**Disclosures:** None

